# Epidemiology of Group B *Streptococcus*: Maternal Colonization and Infant Disease in Kampala, Uganda

**DOI:** 10.1093/ofid/ofaf167

**Published:** 2025-03-18

**Authors:** Mary Kyohere, Hannah Georgia Davies, Konstantinos Karampatsas, Liberty Cantrell, Philippa Musoke, Annettee Nakimuli, Valerie Tusubira, Juliet Sendagala Nsimire, Dorota Jamrozy, Uzma Basit Khan, Stephen D Bentley, Owen B Spiller, Caitlin Farley, Tom Hall, Olwenn Daniel, Simon Beach, Nick Andrews, Stephanie J Schrag, Clare L Cutland, Andrew Gorringe, Stephanie Leung, Stephen Taylor, Paul T Heath, Stephen Cose, Carol Baker, Merryn Voysey, Kirsty Le Doare, Musa Sekikubo, for the PROGRESS Study Group, Abdelmajid Djennad, Agnes Nyamaizi, Agnes Ssali, Alexander Amone, Amusa Wamawobe, Carol Nanyunja, Christine Najuka, Cleophas Komugisha, Christine Sseremba, Lydia Nakibuuka, Daniel Kibirige, Dan R Shelley, Edward A R Portal, Ellie Duckworth, Emilie Karafillakis, Geraldine O'Hara, Godfrey Matovu, Janet Seeley, Joseph Peacock, Katie Cowie, Lauren Hookham, Madeleine Cochet, Margaret Sewegaba, Maxensia Owor, Melanie Etti, Moses Musooko, Patience Atuhaire, Phiona Nalubega, Pooja Ravji, Richard Katungye, Ritah Namugumya, Rosalin Parks, Rose Azuba, Sam Kipyeko, Tim Old, Tobius Mutabazi, Vicki Chalker

**Affiliations:** Makerere University–Johns Hopkins University Research Collaboration, Kampala, Uganda; Centre for Neonatal and Paediatric Infection, Institute of Infection and Immunity, City St George's, University of London, London, United Kingdom; Centre for Neonatal and Paediatric Infection, Institute of Infection and Immunity, City St George's, University of London, London, United Kingdom; Clinical Research Unit, Department of Infectious and Tropical Diseases, London School of Hygiene and Tropical Medicine, London, United Kingdom; Centre for Neonatal and Paediatric Infection, Institute of Infection and Immunity, City St George's, University of London, London, United Kingdom; Oxford Vaccine Group, Department of Paediatrics, University of Oxford, Oxford, United Kingdom; Makerere University–Johns Hopkins University Research Collaboration, Kampala, Uganda; Department of Paediatrics and Child Health, College of Health Sciences, Makerere University, Kampala, Uganda; Department of Obstetrics and Gynaecology, College of Health Sciences, Makerere University, Kampala, Uganda; Makerere University–Johns Hopkins University Research Collaboration, Kampala, Uganda; Clinical Diagnostics Laboratories, MRC/UVRI/LSHTM, Entebbe, Uganda; Parasites and Microbes Programme, Wellcome Sanger Institute, Hinxton, United Kingdom; Parasites and Microbes Programme, Wellcome Sanger Institute, Hinxton, United Kingdom; Parasites and Microbes Programme, Wellcome Sanger Institute, Hinxton, United Kingdom; Division of Infection and Immunity, School of Medicine, Cardiff University, Cardiff, United Kingdom; Division of Infection and Immunity, School of Medicine, Cardiff University, Cardiff, United Kingdom; Centre for Neonatal and Paediatric Infection, Institute of Infection and Immunity, City St George's, University of London, London, United Kingdom; Centre for Neonatal and Paediatric Infection, Institute of Infection and Immunity, City St George's, University of London, London, United Kingdom; Centre for Neonatal and Paediatric Infection, Institute of Infection and Immunity, City St George's, University of London, London, United Kingdom; UK Health Security Agency, London, United Kingdom; National Center for Immunization and Respiratory Diseases, Centers for Disease Control and Prevention, Atlanta, Georgia, USA; Wits African Leadership in Vaccinology Expertise, School of Pathology, Faculty of Health Science, University of the Witwatersrand, Johannesburg, South Africa; Pathogen Immunology Group, UK Health Security Agency, Salisbury, United Kingdom; Pathogen Immunology Group, UK Health Security Agency, Salisbury, United Kingdom; Pathogen Immunology Group, UK Health Security Agency, Salisbury, United Kingdom; Centre for Neonatal and Paediatric Infection, Institute of Infection and Immunity, City St George's, University of London, London, United Kingdom; Medical Research Council/Uganda Virus Research Institute and London School of Hygiene and Tropical Medicine Uganda Research Unit, Entebbe, Uganda; McGovern Medical School, University of Texas Health Science Center, Houston, Texas, USA; Oxford Vaccine Group, Department of Paediatrics, University of Oxford, Oxford, United Kingdom; Centre for Neonatal and Paediatric Infection, Institute of Infection and Immunity, City St George's, University of London, London, United Kingdom; Medical Research Council/Uganda Virus Research Institute and London School of Hygiene and Tropical Medicine Uganda Research Unit, Entebbe, Uganda; Department of Obstetrics and Gynaecology, College of Health Sciences, Makerere University, Kampala, Uganda

**Keywords:** anticapsular antibody, correlate of protection, group B *Streptococcus*, invasive disease, risk reduction

## Abstract

**Background:**

Child survival rates have improved globally, but neonatal mortality due to infections, such as group B *Streptococcus* (GBS), remains a significant concern. The global burden of GBS-related morbidity and mortality is substantial. However, data from low and middle-income countries are lacking. Vaccination during pregnancy could be a feasible strategy to address GBS-related disease burden.

**Methods:**

We assessed maternal rectovaginal GBS colonization and neonatal disease rates in a prospective cohort of 6062 women–infant pairs. Surveillance for invasive infant disease occurred in parallel at 2 Kampala hospital sites. In a nested case-control study, we identified infants <90 days of age with invasive GBS disease (iGBS) (n = 24) and healthy infants born to mothers colonized with GBS (n = 72). We measured serotype-specific anticapsular immunoglobulin G (IgG) in cord blood/infant sera using a validated multiplex Luminex assay.

**Results:**

We found a high incidence of iGBS (1.0 per 1000 live births) within the first 90 days of life across the surveillance sites, associated with a high case fatality rate (18.2%). Maternal GBS colonization prevalence was consistent with other studies in the region (14.7% [95% confidence interval, 13.7%–15.6%]). IgG geometric mean concentrations were lower in cases than controls for serotypes Ia (0.005 vs 0.12 µg/mL; *P* = .05) and III (0.011 vs 0.036 µg/mL; *P* = .07) and in an aggregate analysis of all serotypes (0.014 vs 0.05 µg/mL; *P* = .02).

**Conclusions:**

We found that GBS is an important cause of neonatal and young infant disease in Uganda and confirmed that maternally derived antibodies were lower in early-onset GBS cases than in healthy exposed controls.

## Teaser

We found that group B *Streptococcus* (GBS) is an important cause of neonatal and young infant disease in Uganda and confirmed that maternally derived antibodies were lower in early-onset GBS cases than in healthy exposed controls.

Although child survival rates have improved, neonatal mortality remains a significant concern, with infections being a leading cause of neonatal deaths [1]. *Streptococcus agalactiae* (group B *Streptococcus* [GBS]) is one of the primary causes of sepsis and meningitis in neonates and young infants in most countries [2, 3]⁠. It can manifest as early-onset neonatal disease (EOGBS: 0–6 days) or late-onset disease (LOGBS: 7–89 days).

The global burden of GBS-related morbidity and mortality is substantial, resulting in a high number of cases, infant deaths, stillbirths, and long-term neurodevelopmental impairment [2]. However, data from low- and middle-income countries (LMICs) are limited. A 2017 systematic review identified 90 studies on the incidence of infant invasive GBS disease (iGBS), but only 12 were from Africa [3]. Similarly, data on maternal colonization, the main risk factor for EOGBS, are sparse in African countries [4]. Closing these gaps is essential to informing potential preventive interventions.

In high-income countries (HICs), intrapartum antibiotic prophylaxis (IAP) has significantly reduced the incidence of EOGBS [5]. However, implementing such strategies in LMICs is challenging due to financial and practical constraints and limited access to healthcare facilities [6]. Maternal vaccination during pregnancy may offer a feasible strategy to address GBS-related disease burden, especially in settings where microbiologic screening and IAP are not readily available [7]. The licensure of a GBS vaccine faces a major challenge in that >60 000 pregnant women–infant pairs would need to be enrolled into a prospective clinical trial to demonstrate efficacy [8]. An alternate pathway to licensure could entail demonstrating safety in pregnant women and benchmarking vaccine-induced immunogenicity against correlates of protection defined in seroepidemiological studies [9] with subsequent postlicensure evaluation of effectiveness.

We aimed to estimate the incidence of iGBS in infants and maternal colonization rates in Kampala, Uganda. The nested case-control study aimed to compare antibody concentrations in the cord blood of infants with iGBS to healthy controls.

## METHODS

### Study Design

We undertook a longitudinal, observational study at Kawempe National Referral Hospital (KNRH) in Kampala, Uganda. The fiscal year 2020–2021 gross national income per capita for Uganda is $840, classifying the country as low-income according to the World Bank's global income classification issued on 1 July 2022 [10]. Women consented at the time of birth to their and their infant's participation from birth until 90 days postpartum (birth cohort). In parallel, we monitored hospitalized infants ≤90 days old who were diagnosed with iGBS at KNRH and Mulago National Referral Hospital (MNRH) in Kampala but were not enrolled in the birth cohort (surveillance cohort) [11]. Details of the study setting are described in the Supplementary Methods.

### Birth Cohort

Recruitment took place between 24 April 2019 and 1 September 2020. Eligible participants provided verbal consent to collect samples at delivery (cord blood, separate rectal and vaginal swabs) followed by written informed consent after recovery. If the woman had a stillbirth, consent was requested to collect a heart-blood aspirate, which was used for both culture and measuring anti-GBS capsular polysaccharide (CPS) immunoglobulin G (IgG) concentrations in the serum. Participants received phone follow-ups at 3 time points: within 10 days of the infant's life, 1 month after childbirth, and 90 days postdelivery. Infants from the cohort diagnosed with iGBS (defined as isolation of GBS from cerebrospinal fluid or blood culture) had a serum sample (acute serum) and a rectal swab collected. Women were eligible if they were aged ≥18 years and delivering at Kawempe hospital or if they were emancipated minors aged between 14 and 17 years of age, and were willing to stay in the area for the infant’s first 3 months of life (or willing to travel to the clinic until their child was 2 years old if their infant had known or presumed GBS infection).

### Surveillance Cohort

Recruitment took place between 1 April 2019 and 11 January 2022. Infants aged 0–90 days seeking care at KNRH or MNRH and diagnosed with iGBS were eligible for recruitment into the surveillance cohort. If parents agreed, we collected a serum sample (acute serum) and a rectal swab.

For both the birth cohort and the active surveillance cohort, recruitment was suspended between March 2020 and May 2020 due to lockdown restrictions associated with the coronavirus disease 2019 pandemic.

### Nested Case-Control Study

In a nested case-control study, each eligible patient case was matched to 3 healthy controls (infants born to mothers colonized with the same serotype, not exposed to intrapartum antibiotics as evidenced by clinical history and in vitro testing, and who survived to 90 days of life and were not admitted with an illness between birth and 90 days).

### Laboratory Methods

Rectal and vaginal swabs were collected in Amies transport medium and transported for processing to the Medical Research Council/Uganda Virus Research Institute and London School of Hygiene and Tropical Medicine Research Unit, Entebbe, Uganda. Samples were sent to MRC with a cold chain maintained at the end of each working day and plated on the same day. They were cultured in Todd Hewitt broth and incubated for 24 hours before being subcultured on Chromagar Strep B. Blood and cerebrospinal fluid cultures were processed at Makerere Microbiology Laboratories, Kampala. Detailed laboratory methods have been previously published [11]. Antimicrobial susceptibility testing was performed at Cardiff University (Supplementary Methods). Minimum inhibitory concentrations (MICs) were determined for tetracycline, benzylpenicillin, gentamicin, erythromycin, clindamycin, levofloxacin, and chloramphenicol. High-level gentamicin resistance (HLGR) was defined as gentamicin MIC >128 μg/mL. Extracted genomic DNA using the QiaSymphony method was sent for whole genome sequencing (WGS) at the Wellcome Sanger Institute, Hinxton, United Kingdom (Supplementary Methods). WGS was performed on the Illumina NovaSeq 6000 platform with 150 bp paired-end reads as previously described (Supplementary Methods) [12]. Serotype-specific anti-GBS CPS IgG concentrations for serotypes Ia, Ib, II III, IV, and V were determined using the GASTON-adopted multiplex immunoassay in cord and infant sera (Supplementary Methods 1) [13].

### Statistical Analysis

Demographic and clinical characteristics were presented using proportions for categorical variables and medians for quantitative variables. The prevalence of GBS colonization was expressed as a proportion of the total number of participants in the birth cohort swabbed. The incidence of iGBS in the birth cohort was estimated using denominators of facility births in KNRH. Multivariable logistic regression models were used to assess the association between GBS colonization and pregnancy outcomes as described in the Supplementary Methods. Geometric mean concentrations (GMCs) were reported, and the Mann-Whitney *U* test was used to compare concentrations between cases and controls. For patients with both cord and acute serum collected, cord serum was used. Serotypes with <5 cases were excluded from individual analysis due to insufficient sample size, but were included in the aggregate analysis. Statistical analyses were carried out in R version 4.3.0.

### Ethical Considerations

Ethical approval for this study was granted by the Makerere University College of Health Sciences School of Medicine Research Ethics Committee (reference number 2018-130) and registered with the Uganda National Council for Science and Technology (reference number HS 2496). Ethical approval for this study was also granted by the St George's, University of London Research Ethics Committee (reference number 2020.0024).

## RESULTS

### Recruitment and Patient Characteristics

**Figure 1.**
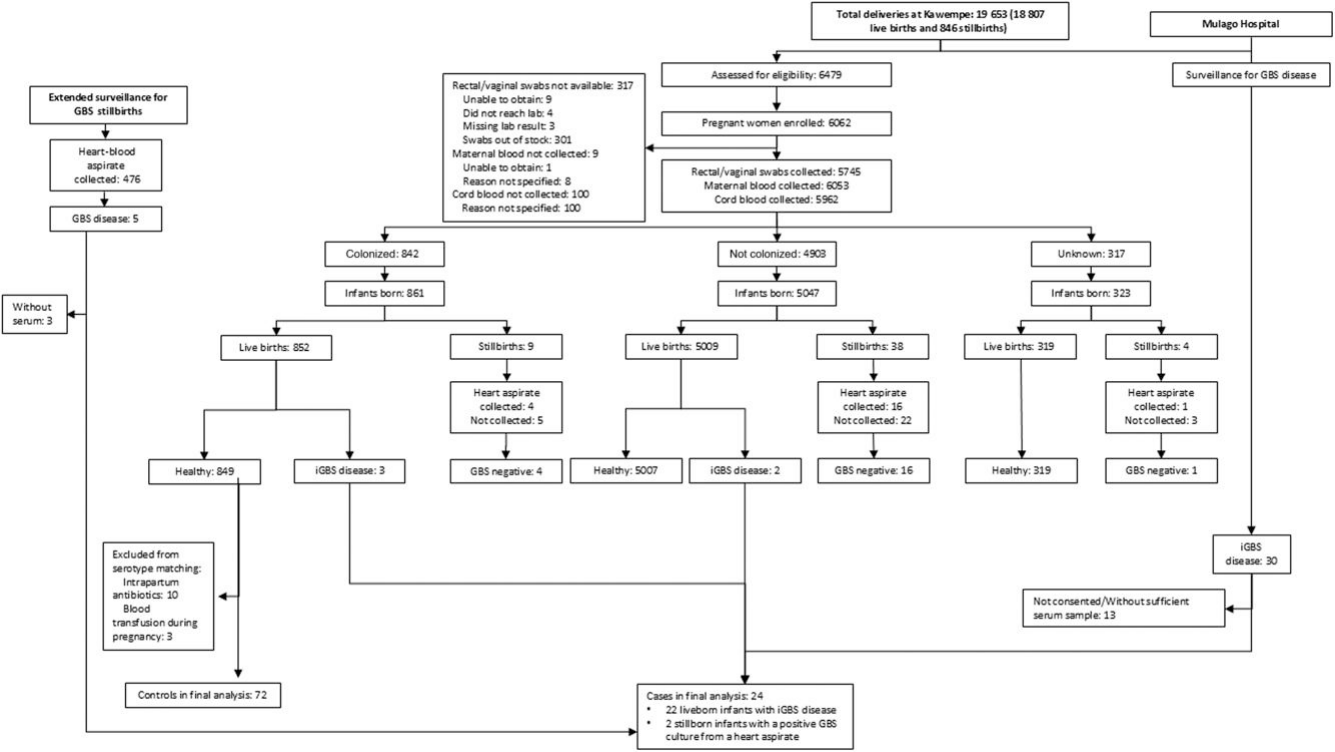
Study cohort diagram. Abbreviations: GBS, group B *Streptococcus*; iGBS, invasive group B *Streptococcus* disease.

There were a total of 19 653 deliveries at KNRH during the birth cohort recruitment study period (18 807 [95.7%] live births and 846 [4.3%] stillbirths). Overall, 6479 of 19 653 (32.9%) women were screened prospectively for eligibility, of whom 6062 (93.6%) were enrolled into the study, resulting in 6170 live births in the birth cohort (Figure 1). The demographic and clinical characteristics of pregnant women and infants are summarized in Tables 1 and 2.

**Table 1.** Demographic and Clinical Characteristics of Enrolled Pregnant Women

Characteristic	Overall (N = 6,062)
Age, y, median (IQR)	25 (21–29)
Age group, y	
<18	157 (2.6)
18–24	2773 (45.7)
25–34	2584 (42.6)
35–41	513 (8.5)
>41	35 (0.6)
Education level	
No formal education	309 (5.1)
Primary	1697 (28.0)
Secondary	3303 (54.5)
Tertiary/university	753 (12.4)
Gravidity	
1	2007 (33.1)
2–4	3165 (52.2)
>4	890 (14.7)
Previous stillbirth among those with previous pregnancy (n = 4055)	
Previous stillbirth	183 (4.5)
No previous stillbirth	3872 (95.5)
Previous abortion among those with previous pregnancy (n = 4055)	
Previous abortion	1099 (27.1)
No previous abortion	2956 (72.9)
Previous child death (<5 y) among those with previous live birth (n = 3697)	
Yes	277 (7.5)
No	3420 (92.5)
Total No. of pregnancy tetanus immunizations	
0	117 (1.9)
1	682 (11.3)
2–4	3265 (53.9)
>4	1946 (32.1)
Unknown	52 (0.8)
Delivery	
Vaginal	5522 (91.1)
Cesarean	461 (7.6)
Assisted vaginal	78 (1.3)
Unknown	1 (<0.1)
Duration of ROM, h	
<18	4787 (79.0)
≥18	785 (12.9)
Unknown	490 (8.1)
Antibiotics during labor	
Yes	116 (1.9)
No	5945 (98.1)
Unknown	1 (<0.1)
Multiple pregnancy	
Singleton	5897 (97.3)
Twins	162 (2.7)
Triplets	3 (<0.1)
Maternal death	3 (<0.1)
Fever during labor	32 (0.5)
HIV status	
Negative	5452 (89.9)
Positive	599 (9.9)
Unknown	11 (0.2)
Syphilis	
Positive	100 (1.6)
Negative	5928 (97.8)
Unknown	34 (0.6)
Hepatitis B (n = 6037)	
Positive	131 (2.2)
Negative	5906 (97.4)
Unknown	25 (0.4)
Maternal nutrition	
Malnourished (MUAC <21 cm)	61 (1.0)
Underweight (MUAC 21 to <23 cm)	147 (2.4)
Normal weight (MUAC 23 to <27 cm)	2787 (46.0)
Overweight (MUAC 27 to <31 cm)	2630 (43.4)
Obese (MUAC ≥31 cm)	435 (7.2)
Unknown	2 (<0.1)
Tobacco/cigarettes	
Currently smokes	8 (0.1)
Has quit smoking during pregnancy	5 (<0.1)
Never smoked	6049 (99.8)
Pipe	
Currently smokes from a pipe	15 (0.2)
Has quit pipe smoking during pregnancy	5 (<0.1)
Never smoked a pipe	6042 (99.7)
Alcohol	
Currently drinks alcohol	336 (5.5)
Has quit drinking alcohol during pregnancy	153 (2.5)
Never drinks alcohol	5573 (92.0)

Data are presented as No. (%) unless otherwise indicated.

Abbreviations: HIV, human immunodeficiency virus; IQR, interquartile range; MUAC, mid-upper arm circumference; ROM, rupture of membranes.

**Table 2.** Demographic and Clinical Characteristics of All Live Newborns and Stillbirths in the Birth Cohort

Characteristic	Overall (N = 6231)
Sex (n = 6229)	
Male	3133 (50.3)
Female	3096 (49.7)
Unknown	2 (<0.1)
HIV exposed (n = 6220)	
Negative	5601 (89.9)
Positive	619 (9.9)
Unknown	11 (0.2)
Birth outcome	
Live born	6170 (99.0)
Stillborn	61 (1.0)
Infant death between birth and 90 d	161 (2.6)
Death in labor ward	12 (7.5)
Death at postnatal ward/NICU	78 (48.4)
Death after hospital discharge	71 (44.1)
Term/preterm (Ballard)	
≥37 wk	5756 (92.4)
≥32 to <37 wk	365 (5.9)
<32 wk	44 (0.7)
Unknown	66 (1.0)
Birth weight, g	
<2500	724 (11.6)
≥2500	5502 (88.3)
Unknown	5 (0.1)
Birth weight <2000 g	222 (3.6)
Resuscitation at birth	
Required resuscitation	721 (11.6)
Did not require resuscitation	5448 (87.4)
Unknown	62 (1.0)
Congenital abnormalities (n = 5846)	
Yes	72 (1.2)
No	5774 (92.6)
Unknown	385 (6.2)

Data are presented as No. (%).

Abbreviations: HIV, human immunodeficiency virus; NICU, neonatal intensive care unit.

### Maternal GBS Colonization

Overall, 5746 of 6062 (94.8%) swabs were collected. Of these, 842 women (14.7% [95% confidence interval {CI}, 13.7%–15.6%]) were colonized with GBS at delivery. GBS colonization was not associated with any of the maternal baseline characteristics modeled (Supplementary Table 1). There was no evidence of an association between maternal GBS colonization and adverse pregnancy outcomes in multivariable models (Supplementary Tables 2–6).

**Figure 2.**
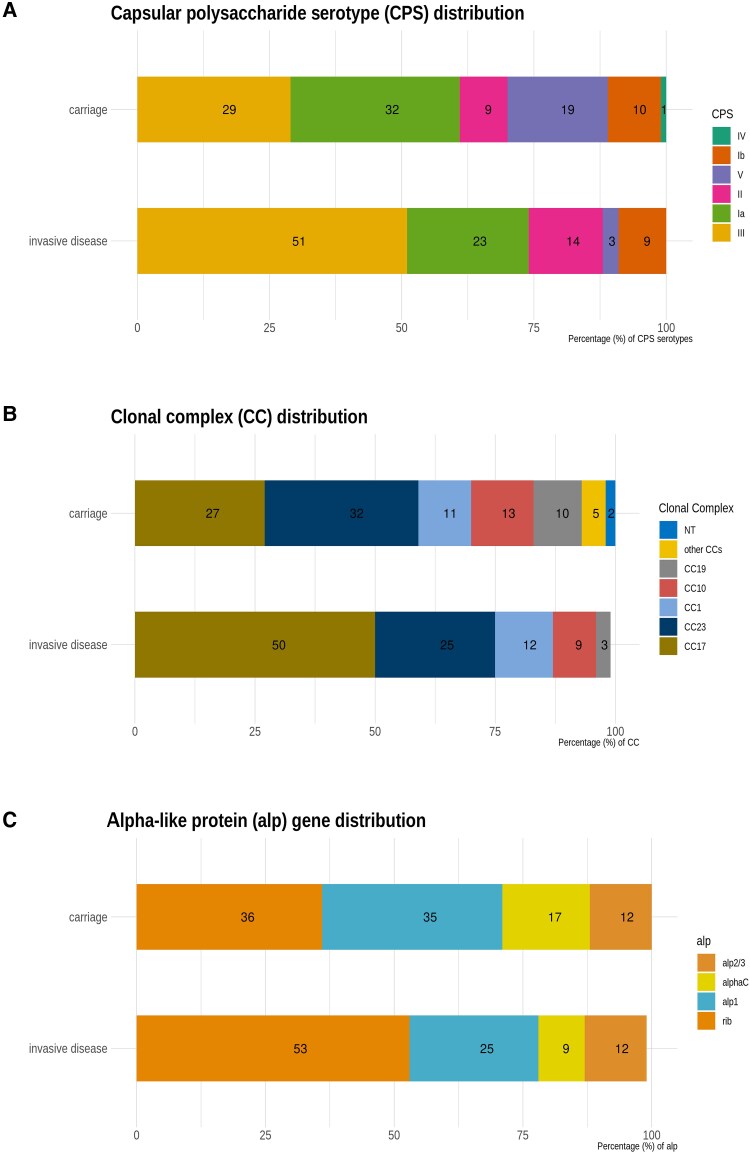
*A*, The percentage of different serotypes in group B *Streptococcus* (GBS) isolates from maternal colonization, and invasive GBS disease. *B*, The percentage of different clonal complexes in GBS isolates from maternal colonization, and invasive GBS disease. *C*, The percentage of different alpha-like protein genes in GBS isolates from maternal colonization, and invasive GBS disease. Abbreviations: alp, alpha-like protein; CC, clonal complex; CPS, capsular polysaccharide; NT, not typed.

CPS serotyping and alpha-like protein (alp) gene analysis from WGS was done on positive GBS swabs from 766 of 842 (91.0%) colonized pregnant women. Serotypes Ia (244/766 [31.9%]) and III (222/766 [29.0%]) were most common among colonizing isolates, followed by serotypes V (142/766 [18.5%]), Ib (76/766 [9.9%]), II (69/766 [9.0%]), IV (10/766 [1.3%]), and VI (1/766 [0.1%]) (Figure 2*A*). The large majority of the isolates (778/766 [93%]) clustered into 5 major GBS clonal complexes (CCs): CC23 (242/766 [31.6%]), CC17 (207/766 [27.0%]), CC10 (103/766 [13.4%]), CC1 (83/766 [10.8%]), and CC19 (73/766 [9.5%]) (Figure 2*B*). A total of 764 of 766 (99.7%) isolates were positive for the presence of an alp gene (alp1, alp2/3, alphaC, and rib). The most prevalent was rib (276/766 [36.0%]), followed by alp1 (267/766 [34.9%]), alphaC (127/766 [16.6%]), and alp2/3 (94/766 [12.3%]) (Figure 2*C*). The CPS serotype and alp gene distribution among the main GBS CCs are shown in Supplementary Figure 1. The alp gene distribution among the CPS serotypes is shown in Supplementary Figure 2.

### Infants With iGBS

A total of 35 cases of iGBS were identified from KNRH and MNRH. Of these, 18 cases involved infants born at KNRH, including 5 from the birth cohort, while 17 involved infants transferred to Mulago from other health centers (Figure 1). The incidence of iGBS within the birth cohort was 0.8 cases per 1000 live births (5/6170). For babies born at KNRH, the incidence rate, based on birth data between 24 April 2019 and 1 September 2020, was 1.0 cases per 1000 live births (18/18 807). Most cases (31/35 [88.6%]) were EOGBS, and almost one-third (11/35 [31.4%]) were born preterm. Among the 4 LOGBS cases (symptom onset on days 8, 14, 21, and 23), 3 were admitted directly from home, while 1 was referred from another health center after initially presenting from home. Follow-up calls did not identify any additional cases. The case fatality rate was 18.2% (Supplementary Table 7).

Among the 35 case patients, 18 (51.4%) had serotype III, 8 (22.9%) had serotype Ia, 5 (14.3%) had serotype II, 3 (8.6%) had serotype Ib, and 1 (2.9%) had serotype V disease (Figure 2*A*). WGS was completed for 32 of 35 cases. All invasive isolates grouped into 5 major GBS CCs: CC17 (16/32 [50.0%]), CC23 (8/32 [25.0%]), CC1 (4/32 [12.5%]), CC10 (3/32 [9.4%]), and CC19 (1/32 [3.1%]) (Figure 2*B*). All isolates tested positive for alp genes, with the most common being rib (17/32 [53.1%]), followed by alp1 (8/32 [25.0%]), alp2/3 (4/32 [12.5%]), and alphaC (3/32 [9.4%]) (Figure 2*C*).

### Antimicrobial Susceptibility

All colonizing isolates were analyzed for the presence of genes associated with antimicrobial resistance (Supplementary Table 8). A total of 739 of 766 (96.5%) carried at least 1 tetracycline resistance gene (tetM or tetO). A total of 207 of 766 (27.0%) carried at least 1 macrolide–lincosamide–streptogramin B (MLSB) resistance gene (ermA, ermB, ermT, lnuB, lsaC, lsaE, mefA, mphC, msrA, or msrD). A total of 12 of 766 (1.6%) harbored the aac(6′)–aph(2″) gene associated with HLGR, and 7 of 766 (0.9%) carried PBP2x transpeptidase sequence variants associated with reduced β-lactam susceptibility. Phenotypic antimicrobial susceptibility testing (AST) results were available for 748 of 766 (97.7%) isolates (Supplementary Table 8). All isolates, including those with PBP2x variants, were susceptible to penicillin. A total of 5 of 748 isolates (0.7%) had a benzylpenicillin MIC of 0.125 mg/L, but none exceeded 0.25 mg/L. HLGR was observed in 17 of 748 (2.3%) isolates. There was some discordance between genotype and phenotype for erythromycin resistance, with 207 of 766 (27.0%) isolates showing genotypic resistance compared to 288 of 748 (38.5%) displaying phenotypic resistance. Phenotypic resistance was found in 732 of 748 (97.9%) isolates for tetracycline, and 39 of 748 (5.2%) for levofloxacin.

WGS was completed for 32 of 35 cases (Supplementary Table 8). All 32 invasive isolates carried at least 1 tetracycline resistance gene and 13 of 32 (40.6%) carried at least 1 MLSB resistance gene. None of the invasive isolates carried PBP2x variants. Phenotypic AST results were available for 32 of 35 isolates (Supplementary Table 8). All 32 isolates were susceptible to penicillin and clindamycin, and none exhibited HLGR. Phenotypic resistance was observed in 31 of 32 isolates (96.9%) for tetracycline, 15 of 32 (46.9%) for erythromycin, and 1 of 32 (3.1%) for levofloxacin.

The prevalence of MLSB resistance genes among invasive and colonizing isolates was higher in CC1 (79/83 [95.2%]) and CC19 (51/73 [69.9%]) and lower in CC23 (7/242 [2.9%]) and CC17 (29/207 [14.0%]). MLSB resistance genes were most prevalent among isolates with serotype V (114/143 [79.7%]) and less common among isolates with serotype Ia (16/252 [6.3%]) and III (39/240 [16.2%]) (Supplementary Figure 3).

### Anti-CPS IgG Concentration in Infants With iGBS Compared to Healthy Controls

**Figure 3.**
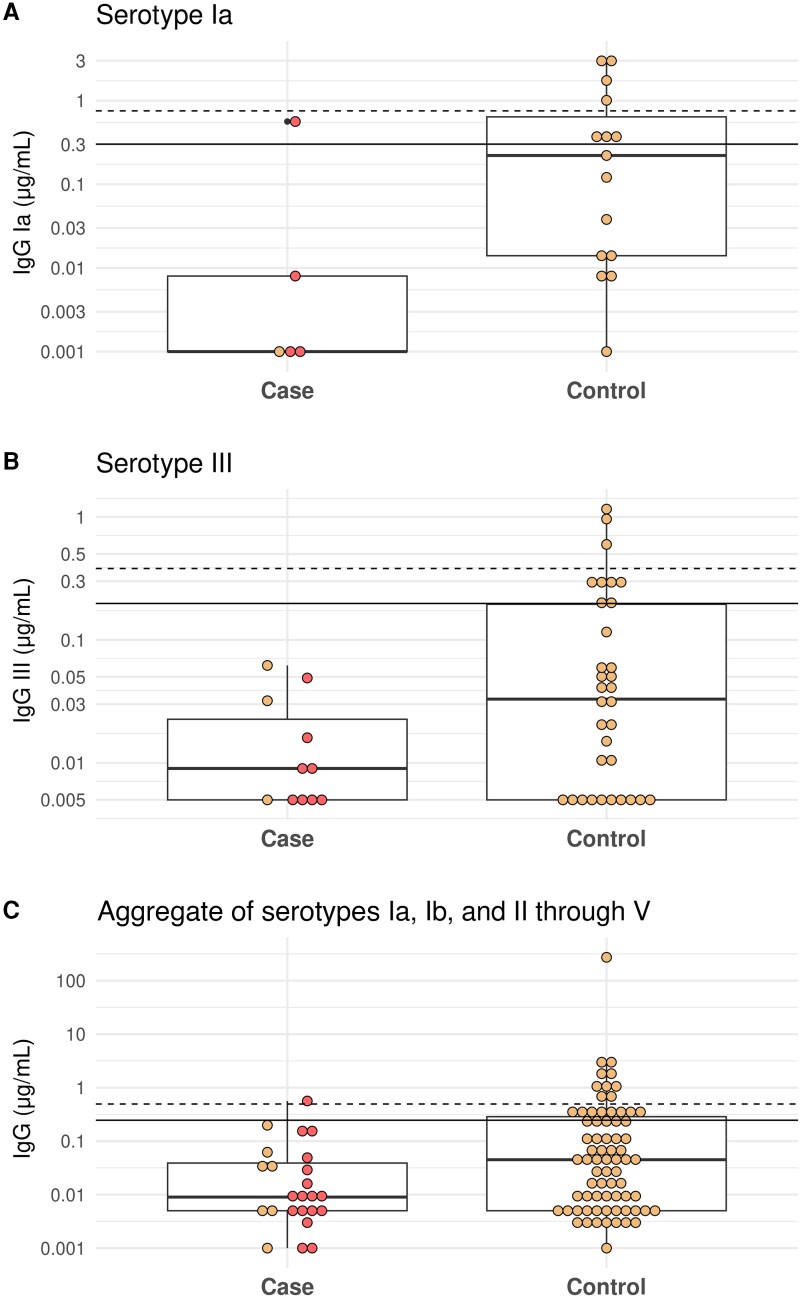
*A*, Anti–capsular polysaccharide (CPS) immunoglobulin G (IgG) concentrations among infant case patients and controls for serotype Ia. *B*, Anti-CPS IgG concentrations among infant case patients and controls for serotype III. *C*, Anti-CPS IgG concentrations among infant case patients and controls for aggregated serotypes. The horizontal continuous line indicates the recently proposed threshold associated with 80% risk reduction. The horizontal dashed line indicates the recently proposed threshold associated with 90% risk reduction. Each point represents an individual sample. Yellow points indicate cord serum, and red points indicate infant serum collected during the acute phase of the disease.

The demographic and clinical characteristics of the case and control groups are summarized in Table 3. Case patients were more likely to be born preterm and with low birth weight. Among the 24 infants with enough sera to be included in the case group, 19 of 24 (79.2%) were diagnosed with EOGBS within 0–2 days of birth, 3 of 24 (12.5%) developed LOGBS with symptom onset on days 8, 14, and 23, respectively, and 2 of 24 (8.3%) were antepartum stillbirths born at term. Cord blood samples were available for 7 of 24 (29.2%) cases (4 with EOGBS, 1 with LOGBS, and 2 stillbirths). Acute disease sera were collected from 19 of 24 (79.2%) cases, with a median of 6 days (range, 3–8 days) between symptom onset and serum collection. For serotype Ia, controls had higher GMCs of anti-CPS IgG compared to the case patients (0.12 vs 0.005 µg/mL; *P* = .05) (Figure 3*A*). For serotype III, controls had higher GMCs than case patients (0.036 vs 0.011 µg/mL; *P* = .07) (Figure 3*B*). In an aggregate analysis of all serotypes, controls had higher GMCs of anti-CPS IgG than case patients (0.05 vs 0.014 µg/mL; *P* = .02) (Figure 3*C*). When considering only EOGBS cases, controls had marginally higher GMCs than case patients for serotype Ia (0.12 vs 0.005 µg/mL; *P* = .05) and serotype III (0.036 vs 0.010 µg/mL; *P* = .05). The difference was statistically significant when all serotypes were combined (0.05 vs 0.011 µg/mL; *P* = .02) (Supplementary Figure 4).

**Table 3.** Demographic and Clinical Characteristics of Patient Cases and Controls

Characteristic	Cases (n = 24)	Controls (n = 72)	*P* Value^a^
Serotype distribution			
Ia	5 (20.8)	15 (20.8)	^b^
Ib	3 (12.5)	9 (12.5)	
II	4 (16.7)	12 (16.7)	
III	11 (45.8)	33 (45.8)	
V	1 (4.2)	3 (4.2)	
Onset of disease, days of age		NA	
Stillbirth	2 (8.3)		
0	10 (41.6)		
1	8 (33.3)		
2	1 (4.2)		
3–6	0 (0.0)		
7–13	1 (4.2)		
14–20	1 (4.2)		
>20	1 (4.2)		
Gestational age at birth, wk	37 (32.5–38.5)	39 (38–40)	.002
Term (≥37)	17 (70.8)	69 (95.8)	
Preterm (<37)	7 (29.2)	3 (4.2)	
34 to <37	2 (8.3)	2 (2.8)	
32 to <34	3 (12.5)	1 (1.4)	
28 to <32	2 (8.3)	0 (0.0)	
Sex			.6
Male	12 (50.0)	40 (55.6)	
Female	12 (50.0)	32 (44.4)	
Birth weight, g	2585 (2120–3350)	3240 (2925–3580)	<.001
Low birth weight (<2500 g)	11 (45.8)	1 (1.4)	
Normal birth weight (≥2500 g)	13 (54.2)	71 (98.6)	
Maternal HIV status			1.0
Living with HIV	2 (8.3)	5 (6.9)	
Not living with HIV	22 (91.7)	67 (93.1)	
Antibiotics during labor			1.0
No	24 (100.0)	72 (100.0)	
Yes	0 (0.0)	0 (0.0)	

Data are presented as No. (%) or median (interquartile range) unless otherwise indicated.

Abbreviations: HIV, human immunodeficiency virus; NA, not applicable.

^a^Fisher exact test.

^b^ *P* value for serotype distribution is missing as the study is matched for 1 case to 3 controls.

## DISCUSSION

This study provides the first estimates of iGBS incidence in newborns and young infants in Kampala, Uganda. For the first time, it also compares serum anti-GBS CPS IgG levels between Ugandan infants with iGBS and healthy controls born to women colonized with GBS at delivery. Our data confirmed that GBS is an important cause of neonatal and young infant disease in Uganda [14]. In HICs, where there is good capture of cases and routine laboratory surveillance, GBS has been well-recognized as a leading cause of early- and late-onset disease in neonates and young infants since the 1990s [15]. However, there is still uncertainty about the incidence of iGBS in LMICs. Well-conducted studies in healthcare facilities in South Africa, Kenya, and The Gambia have indicated that GBS is an important neonatal pathogen in those settings [16–18]. Our study confirms these findings, reporting a disease incidence similar to the previous estimate of iGBS in Africa (1.0 vs 1.12 per 1000 live births) [19]. The case fatality ratio in our cohort was high, in keeping with previous regional estimates [2].

The maternal colonization rate was 14.7% (95% CI, 13.7%–15.6%), which is lower than the 28.8% reported in a 2016 study at Mbarara Regional Referral Hospital in rural southwestern Uganda [20]. However, it is consistent with the most recent region-specific GBS colonization prevalence for sub-Saharan Africa, estimated at 16.1% (95% CI, 13.7%–19.0%) [2]. These are high-quality data, given that the study used the gold-standard sampling method: rectal/vaginal swabs collected at delivery with selective enrichment in the laboratory, addressing some of the biases noted in earlier studies [4]. It is also important to highlight that data from Africa outside of South Africa have been sparse, making this estimate from East Africa particularly valuable.

Overall, a hexavalent vaccine (serotypes Ia, Ib, II, III, IV, and V) and an alp-based vaccine would provide comprehensive cover against invasive (100% and 100%, respectively) and colonizing GBS strains (99.7% and 99.7%, respectively) in our cohort. This aligns with a previous systematic review indicating that 93%–99% of iGBS in Africa could potentially be prevented by a hexavalent or alp-based vaccine [21]. Additionally, a large multicountry colonization study found that a hexavalent vaccine would cover 97.3% of all maternal colonizing isolates [22]. While 6 main serotypes are most common, some rarer serotypes have also been identified in Ghana and Egypt [23].

Reassuringly, all invasive and colonizing isolates were susceptible to penicillin. This is of clinical importance, given that penicillin is the first-line drug for treatment and prevention of GBS infections, which is now jeopardized by the emergence of GBS with reduced penicillin susceptibility in HICs and LMICs [24, 25]. In contrast, high rates of phenotypic resistance to erythromycin were observed in both colonizing and invasive isolates. Resistance to erythromycin in GBS strains has risen dramatically in recent years, with rates varying according to geographical regions between 22.5% and 74.1% [26]. The 2 CCs with the highest prevalence of MLSB resistance genes in this and previous studies, CC1 and CC19, could be potential reservoirs of high-risk lineages and require further genomic surveillance [12].

Serotype Ia and III anti-GBS CPS IgG levels were higher in the cord blood of healthy babies born to colonized women compared to those with EOGBS due to the same serotype. Although the difference was small, likely due to the small sample size in our study, these findings are in line with previous research in this area [27–31]. In our aggregate analysis of all serotypes, only 1 case had IgG concentrations above the threshold associated with a 90% risk reduction, as proposed in a seroepidemiological study from South Africa that used a similar design and laboratory methods [32]. However, a key difference between the studies is the proportion of LOGBS cases—12.5% in this study compared to 48% in the South African study. This may be due to case ascertainment in our highly mobile population.

Our study has several limitations. First, the birth cohort included only a third of the total pregnancies in Kawempe, which limits the generalizability of our results to the entire catchment population. However, our careful review of hospital logs enables us to have relative certainty that the women recruited are typical of those presenting to the hospital and those presenting to public hospitals across Uganda. Second, the very low proportion of LOGBS cases suggests a recruitment bias, possibly indicating underascertainment of cases in the community or outpatient settings due to limited access to care for infants not included in the birth cohort during the study. Also, because lumbar punctures are infrequently undertaken in this population, it is possible that GBS meningitis, the main clinical presentation of LOGBS, may have been significantly underestimated. Third, the lower IgG concentrations observed in cases compared to controls may have been confounded by the higher proportion of preterm births. Due to the small sample size, a subanalysis of term births could not be performed. Fourth, since we did not have cord blood samples from all cases, we measured IgG concentrations in acute disease sera. This method is likely a reliable surrogate for EOGBS cases, which formed the vast majority of cases in our study, but it may not be as accurate for LOGBS due to the decline in antibodies after birth. Fifth, due to the small number of case patients, we did not determine the serotype-specific or serotype-aggregate disease risk–IgG concentration relationships. To address the issue of small sample size, we propose to combine the antibody data with that derived from cases and controls from several African and European countries (Malawi, United Kingdom, Netherlands, Italy, and France) and process using the same assay, in order to determine anti-CPS IgG concentration thresholds associated with reduced risk of disease [33]. Finally, we cannot exclude the possibility that we did not capture all infants with infections in the first 3 months of life who were born at 1 of our study sites. To minimize this, in addition to regular telephone follow-up, we reviewed all admission records and compared these to birth records to ensure that case capture was as accurate as possible.

In conclusion, GBS is an important, potentially preventable cause of neonatal disease and death in urban Uganda. Maternal GBS vaccination provides a key opportunity to reduce morbidity and mortality in this high-burden region.

## Supplementary Material

ofaf167_Supplementary_Data

## Supplementary Data

Supplementary materials are available at *Open Forum Infectious Diseases* online. Consisting of data provided by the authors to benefit the reader, the posted materials are not copyedited and are the sole responsibility of the authors, so questions or comments should be addressed to the corresponding author.

## Notes

***Acknowledgments.*** We thank Abdelmajid Djennad, Agnes Nyamaizi, Agnes Ssali, Alexander Amone, Amusa Wamawobe, Carol Nanyunja, Christine Najuka, Cleophas Komugisha, Christine Sseremba, Lydia Nakibuuka, Daniel Kibirige, Dan R. Shelley, Edward A. R. Portal, Ellie Duckworth, Emilie Karafillakis, Geraldine O’Hara, Godfrey Matovu, Janet Seeley, Joseph Peacock, Katie Cowie, Lauren Hookham, Madeleine Cochet, Margaret Sewegaba, Maxensia Owor, Melanie Etti, Moses Musooko, Patience Atuhaire, Phiona Nalubega, Pooja Ravji, Richard Katungye, Ritah Namugumya, Rosalin Parks, Rose Azuba, Sam Kipyeko, Tim Old, Tobius Mutabazi, and Vicki Chalker, for all their contributions.

***Financial support**.* This work was funded by the Gates Foundation (grant numbers OPP1189053 and INV-010426) and the European and Developing Countries Clinical Trials Partnership 2 program supported by the European Union (grant number RIA2018V-2304-PREPARE). K. L. D. is supported by the UK Research and Innovation Future Leaders Fellowship (MR/S016570/1).
