# Supplementary material for: Epidemiology of Group B Streptococcus: Maternal Colonization and Infant Disease in Kampala, Uganda
Source: Open Forum Infect Dis. 2025 Mar 18;12(4):ofaf167. doi: 10.1093/ofid/ofaf167 (PMC11977330; doi:10.1093/ofid/ofaf167)
Supplement: ofaf167_Supplementary_Data [file ofaf167_supplementary_data.zip › Supplementary Material_250303_clean.docx]

**Supplementary Material**

**S1 Methods: Study setting, antimicrobial susceptibility testing, whole Genomic Sequencing, assembly and MLST typing methods and genomic analyses**

**Study Setting**

Kawempe National Referral Hospital is a busy government-funded national referral hospital in Kampala. The hospital performs approximately 21,000 deliveries per year. It provides routine antenatal care for pregnant women, as well as receiving referrals from surrounding health centres for women with high-risk pregnancies or complications. There is a neonatal unit admitting preterm neonates as well as those with birth-related complications, sepsis or congenital anomalies (approximately 11,000 admissions per year).

Mulago National Referral Hospital is a large hospital in Kampala with an official bed capacity of 1790. The hospital's children's services include outpatient appointments, acute admissions, and several inpatient wards. In Uganda, women presenting in labour are managed by midwives according to WHO guidelines for prevention of vertical transmission of infection. Intrapartum antibiotic prophylaxis is not routine and is only provided if women present with fever in labour. ^1^

**Antimicrobial susceptibility testing**

Antimicrobial susceptibility testing was performed at Cardiff University. Single colonies from overnight culture on Colombia blood agar of frozen archived isolates were taken and resuspended to prepare 0.5 McFarland standards in sterile 3mL 0.85% saline (Oxoid, UK) for antimicrobial sensitivity testing. Minimum inhibitory concentrations (MICs) were determined for tetracycline, benzylpenicillin, gentamicin, erythromycin, clindamycin, levofloxacin and chloramphenicol. Plates were inoculated using a multipoint inoculator (MAST URI®DOT) post-autoclave sterilisation of the pins. Inoculated plates were incubated at 37°C for 18-24 hours. Susceptibility was determined according to the European Committee on Antimicrobial Susceptibility Testing (EUCAST) guidelines unless unavailable where Epidemiological Cut off Values (ECOFF) or EUCAST determined PK/PD values were used.

**DNA extraction**

Genomic DNA was extracted using Qiagen DNeasy Blood & Tissue kit (Qiagen, Germany), following the manufacturer's instructions for gram-positive bacteria with modifications by resuspending the overnight bacterial pellet in 44 µL of an enzyme mix containing 20 µL mutanolysin (3,000 U/mL), 20 µL lysozyme (100 mg/mL) and 2 µL RNaseA (Qiagen, Germany) (100 mg/mL) per sample and incubated at 37°C for 2 hours. The subsequent steps were as per the gram-positive bacteria protocol. ^2^

**Whole genome sequencing**

The bacterial genomic DNA was extracted using Qiagen's QIAcube HT system. Library construction was carried out using the 'NEB Ultra II custom kit' on an Agilent Bravo WS automation system. Whole-genome sequencing was performed on the Illumina NovaSeq 6000 platform with 150 bp paired-end reads. Annotated assemblies were produced using a previously described pipeline. ^3^ For each sample, sequence reads were used to create multiple assemblies using VelvetOptimiser v2.2.51 and Velvet v1.2. ^4^ An assembly improvement step was applied to the assembly with the best N50, and contigs were scaffolded using SSPACE, ^5^ with sequence gaps filled using GapFiller. ^6^ Automated annotation was performed using PROKKA v1.11 ^7^ and a Streptococcus-specific database from RefSeq. ^8^

Sequence type and serotype were determined from the whole genome sequence data using the GBS Typer pipeline (https://github.com/sanger-bentley-group/GBS-Typer-sanger-nf). Isolates were assigned to a clonal complex using the geoBURST algorithm in PHYLOVIZ v2.0 and a single locus variant for group definition. ^9^ The sequence data has been deposited in the European Nucleotide Archive (ENA) under study accession PRJEB51700.

**Serological analysis**

We measured serotype-specific anti-GBS CPS IgG concentrations using the GASTON-adopted multiplex immunoassay (MIA) in cord and infant sera ^10^. The GASTON MIA measures anti-CPS antibodies in human serum samples specific to GBS serotypes Ia, Ib, II III, IV and V. The assay is based on Luminex technology; samples were incubated overnight with a 6-plex pool of beads, with each bead region coupled to either serotype Ia, Ib, II, III, IV, or V polysaccharide poly-L-lysine conjugates (Pfizer, Inc.). Samples were diluted to 1/500, 1/5,000, and 1/50,000. Each plate included an 11-point standard curve of multivalent vaccinee reference serum (Pfizer Inc, New York, NY, USA) serially diluted 2.5-fold, and two wells containing assay buffer acted as blank controls ^11^.  Plates were incubated overnight at 4^o^C, shaking at 300RPM (Thermo Solaris, Thermo Fisher Scientific Inc, MA, USA). The next day, the plates were washed with 200 µL of phosphate-buffered saline (PBS)/Tween (1xPBS/0.05%Tween/0.02%/Sodium Azide, pH 7.2) using a plate washer (Tecan Hydrospeed, Tecan, Reading, UK) with a magnetic base to retain the beads. Secondary antibody was added (R-Phycoerythrin Goat Anti-Human IgG Fcy specific, Jackson Laboratories 109-115-098, Jackson ImmunoResearch Ely, UK), at 1/500 (50 µL) to each well, and the plates were incubated for 90 (±15) minutes at room temperature under constant shaking (300 RPM). Following the incubation, the plates were washed again, and 100 µL of wash buffer added to each well. The plates were read on a Bio-plex 200 (Bio-Rad Laboratories, Hercules, CA, USA) at high RP1 (high photomultiplier tube voltage). Data were captured as median fluorescent intensities (MFI) and converted to mcg/mL antibody concentrations using the reference standard curve and accounting for the serum dilution factor. The standardised lower limit of quantitation (LLOQ) values were published recently ^12^**.**

**Statistical analysis**

To assess the association between GBS carriage during pregnancy and pregnancy outcomes, multivariable logistic regression models were used to model the following outcomes: prolonged rupture of membranes defined as rupture of membranes lasting at least 18 hours (PROM), stillbirth, preterm birth <34 weeks (to account for uncertainty around the Ballard score), low birth weight, (defined as less than 2500g), and infant death. Models were adjusted for maternal age, HIV status, maternal nutrition, smoking status, alcohol consumption, and antibiotics given during labour. The models included only participants with complete data, excluding those with any missing variables.

**Table S1: A**ssociation between baseline characteristics and maternal Group B streptococcus (GBS) colonization at delivery

| **Characteristic** | **N maternal samples** | **N positive for GBS** | **OR^1^** | **95% CI^1^** |
| --- | --- | --- | --- | --- |
| **Age of mother (per 1 year of age)** | 5,733 | 840 | 1.01 | 0.99, 1.02 |
| **HIV positive** |  |  |  |  |
| No | 5,183 | 771 | — | — |
| Yes | 550 | 69 | 0.81 | 0.62, 1.05 |
| **Maternal nutrition** |  |  |  |  |
| Normal weight | 2,646 | 369 | — | — |
| Malnourished | 57 | 10 | 1.33 | 0.66, 2.65 |
| Underweight | 139 | 22 | 1.17 | 0.73, 1.87 |
| Overweight | 2,470 | 376 | 1.10 | 0.94, 1.28 |
| Obese | 421 | 63 | 1.06 | 0.79, 1.42 |
| **Parity (per live birth)** | 5,733 | 840 | 1.00 | 0.93, 1.07 |
| **Smokes (tobacco or pipe)** |  |  |  |  |
| No | 5,715 | 837 | — | — |
| Yes | 18 | 3 | 1.20 | 0.34, 4.17 |
| **Drinks alcohol** |  |  |  |  |
| No | 5,418 | 794 | — | — |
| Yes | 315 | 46 | 0.98 | 0.71, 1.36 |

*^1^OR = Odds Ratio, CI = Confidence Interval*

*The outcome for the model is positive GBS swab (vs negative). Those with missing data were excluded from the model.*

*The model includes only those mothers who had no missing data for any predictor variable included in the model, therefore the number of maternal samples is fewer than the number of women enrolled in the study.*

**Table S2:** Association between baseline characteristics and prolonged rupture of membranes.

| **Characteristic** | **N pregnancies** | **N with PROM** | **OR^1^** | **95% CI^1^** |
| --- | --- | --- | --- | --- |
| **GBS status** |  |  |  |  |
| Negative | 4,499 | 641 | — | — |
| Positive | 779 | 106 | 0.96 | 0.77, 1.20 |
| Unknown | 280 | 35 | 0.85 | 0.59, 1.23 |
| **Age of mother (per 1 year of age)** | 5,558 | 782 | 1.0 | 0.98, 1.01 |
| **HIV positive** |  |  |  |  |
| No | 4,999 | 710 | — | — |
| Yes | 559 | 72 | 0.90 | 0.69, 1.17 |
| **Antibiotics in labour** |  |  |  |  |
| No | 5,448 | 744 | — | — |
| Yes | 110 | 38 | 3.47 | 2.31, 5.20 |
| **Drinks alcohol** |  |  |  |  |
| No | 5,245 | 751 | — | — |
| Yes | 313 | 31 | 0.65 | 0.44, 0.95 |
| **Smokes (tobacco or pipe)** |  |  |  |  |
| No | 5,542 | 778 | — | — |
| Yes | 16 | 4 | 2.02 | 0.62, 6.58 |
| **Maternal nutrition** |  |  |  |  |
| Normal weight | 2,591 | 405 | — | — |
| Malnourished | 58 | 11 | 1.25 | 0.64, 2.44 |
| Underweight | 130 | 26 | 1.30 | 0.83, 2.03 |
| Overweight | 2,379 | 293 | 0.77 | 0.65, 0.90 |
| Obese | 400 | 47 | 0.74 | 0.53, 1.02 |

*^1^OR = Odds Ratio, CI = Confidence Interval.*

*The outcome in the model is PROM= yes (vs no). Those with missing data were excluded.*

*The model includes only those mothers who had no missing data for any key variable included in the model, therefore the number of pregnancies is fewer than the number of women enrolled in the study.*

**Table S3:** Association between baseline characteristics and infant death or stillbirth

| **Characteristic** | **N pregnancies** | **N at least one stillbirth or infant death** | **OR^1^** | **95% CI^1^** |
| --- | --- | --- | --- | --- |
| **GBS status** |  |  |  |  |
| Negative | 4,231 | 115 | — | — |
| Positive | 732 | 16 | 0.79 | 0.47, 1.35 |
| Unknown | 266 | 5 | 0.69 | 0.28, 1.71 |
| **Age of mother (per 1 year of age)** | 5,229 | 136 | 1.03 | 0.99, 1.07 |
| **HIV positive** |  |  |  |  |
| No | 4,695 | 123 | — | — |
| Yes | 534 | 13 | 0.90 | 0.50, 1.63 |
| **Antibiotics in labour** |  |  |  |  |
| No | 5,132 | 133 | — | — |
| Yes | 97 | 3 | 1.19 | 0.37, 3.84 |
| **Parity (per live birth)** | 5,229 | 136 | 0.86 | 0.73, 1.02 |
| **Drinks alcohol** |  |  |  |  |
| No | 4,938 | 127 | — | — |
| Yes | 291 | 9 | 1.23 | 0.62, 2.46 |
| **Maternal nutrition** |  |  |  |  |
| Normal weight | 2,439 | 67 | — | — |
| Malnourished | 52 | 1 | 0.70 | 0.10, 5.16 |
| Underweight | 121 | 3 | 0.88 | 0.27, 2.84 |
| Overweight | 2,243 | 58 | 0.94 | 0.66, 1.36 |
| Obese | 374 | 7 | 0.67 | 0.30, 1.49 |
| **Previous infant death (up to 1 year)** |  |  |  |  |
| No | 5,044 | 128 | — | — |
| Yes | 185 | 8 | 2.02 | 0.95, 4.30 |
| **PROM** | 5,229 | 136 | 0.96 | 0.59, 1.58 |

*^1^OR = Odds Ratio, CI = Confidence Interval.*

*The outcome in the model is pregnancy resulting in at least one stillbirth or infant death (vs. no stillbirth or death). Those with missing data were excluded.*

*The model includes only those mothers who had no missing data for any key variable included in the model, therefore the number of pregnancies is fewer than the number of women enrolled in the study.*

**Table S4:** Association between baseline characteristics and preterm birth <34 weeks gestational age. Any infants with Ballard score >= 34 were analysed as not preterm to account for possible inaccuracies in the Ballard score.

| **Characteristic** | **N pregnancies** | **N preterm <34 weeks** | **OR^1^** | **95% CI^1^** |
| --- | --- | --- | --- | --- |
| **GBS status of mother** |  |  |  |  |
| Negative | 4,893 | 78 | — | — |
| Positive | 840 | 9 | 0.68 | 0.34, 1.37 |
| Unknown | 316 | 3 | 0.58 | 0.18, 1.84 |
| **Age of mother (per 1 year of age)** | 6,049 | 90 | 0.95 | 0.91, 0.99 |
| **HIV exposed** |  |  |  |  |
| No | 5,450 | 72 | — | — |
| Yes | 599 | 18 | 2.80 | 1.63, 4.81 |
| **Mother drinks alcohol** |  |  |  |  |
| No | 5,713 | 85 | — | — |
| Yes | 336 | 5 | 1.13 | 0.45, 2.82 |
| **Maternal nutrition** |  |  |  |  |
| Normal weight | 2,784 | 51 | — | — |
| Malnourished | 61 | 2 | 1.66 | 0.39, 7.02 |
| Underweight | 147 | 4 | 1.41 | 0.50, 3.98 |
| Overweight | 2,623 | 28 | 0.66 | 0.41, 1.05 |
| Obese | 434 | 5 | 0.83 | 0.32, 2.12 |
| **Previous miscarriage** |  |  |  |  |
| No | 4,953 | 81 | — | — |
| Yes | 1,096 | 9 | 0.55 | 0.28, 1.12 |

*^1^OR = Odds Ratio, CI = Confidence Interval*

*The outcome in the model is pregnancy resulting in preterm birth (vs. not preterm), where preterm birth was defined as <34 weeks gestational age. Those with missing data were excluded.*

*The model includes only those mothers who had no missing data for any key variable included in the model, therefore the number of pregnancies is fewer than the number of women enrolled in the study.*

**Table S5:** Association between baseline characteristics and low birth weight <2500 g.

| **Characteristic** | **N pregnancies** | **N low birth weight** | **OR^1^** | **95% CI^1^** |
| --- | --- | --- | --- | --- |
| **GBS status of mother** |  |  |  |  |
| Negative | 4,892 | 546 | — | — |
| Positive | 840 | 72 | 0.77 | 0.59, 1.01 |
| Unknown | 316 | 35 | 1.01 | 0.70, 1.47 |
| **Age of mother (per 1 year of age)** | 6,048 | 653 | 0.98 | 0.97, 1.00 |
| **HIV exposed** |  |  |  |  |
| No | 5,449 | 559 | — | — |
| Yes | 599 | 94 | 1.59 | 1.23, 2.06 |
| **Mother drinks alcohol** |  |  |  |  |
| No | 5,712 | 609 | — | — |
| Yes | 336 | 44 | 1.36 | 0.96, 1.92 |
| **Maternal nutrition** |  |  |  |  |
| Normal weight | 2,783 | 344 | — | — |
| Malnourished | 61 | 12 | 1.64 | 0.83, 3.24 |
| Underweight | 147 | 24 | 1.31 | 0.81, 2.11 |
| Overweight | 2,623 | 241 | 0.76 | 0.64, 0.92 |
| Obese | 434 | 32 | 0.61 | 0.41, 0.91 |
| **Infant born preterm** |  |  |  |  |
| No | 5,958 | 580 | — | — |
| Yes | 90 | 73 | 36.1 | 21.1, 61.8 |
| **Antibiotics during labour** |  |  |  |  |
| No | 5,932 | 634 | — | — |
| Yes | 116 | 19 | 1.38 | 0.80, 2.38 |

*^1^OR = Odds Ratio, CI = Confidence Interval*

*The outcome in the model is pregnancy with at least one infant with a birth weight less than 2500g (vs no infants with a low birth weight). Those with missing data were excluded.*

*The model includes only those mothers who had no missing data for any key variable included in the model, therefore the number of pregnancies is fewer than the number of women enrolled in the study.*

**Table S6:** Association between maternal antenatal Group B streptococcus (GBS) colonisation and any adverse pregnancy outcome (defined as any of the following: prolonged rupture of membranes, maternal death, stillbirth, infant death, preterm birth, low birth weight).

| **Characteristic** | **N pregnancies** | **N adverse outcomes** | **OR^1^** | **95% CI^1^** |
| --- | --- | --- | --- | --- |
| **GBS status** |  |  |  |  |
| Negative | 4,892 | 1,179 | — | — |
| Positive | 840 | 179 | 0.86 | 0.72, 1.03 |
| Unknown | 316 | 70 | 0.89 | 0.67, 1.17 |
| **Age of mother (per 1 year of age)** | 6,048 | 1,428 | 0.99 | 0.98, 1.00 |
| **HIV positive** |  |  |  |  |
| No | 5,449 | 1,270 | — | — |
| Yes | 599 | 158 | 1.21 | 1.00, 1.48 |
| **Antibiotics in labour** |  |  |  |  |
| No | 5,932 | 1,380 | — | — |
| Yes | 116 | 48 | 2.35 | 1.61, 3.43 |
| **Drinks alcohol** |  |  |  |  |
| No | 5,712 | 1,353 | — | — |
| Yes | 336 | 75 | 0.96 | 0.73, 1.25 |
| **Smokes (tobacco or pipe)** |  |  |  |  |
| No | 6,029 | 1,421 | — | — |
| Yes | 19 | 7 | 1.75 | 0.67, 4.54 |
| **Maternal nutrition** |  |  |  |  |
| Normal weight | 2,783 | 747 | — | — |
| Malnourished | 61 | 25 | 1.85 | 1.10, 3.10 |
| Underweight | 147 | 45 | 1.16 | 0.81, 1.67 |
| Overweight | 2,623 | 530 | 0.70 | 0.62, 0.80 |
| Obese | 434 | 81 | 0.66 | 0.51, 0.85 |

*^1^OR = Odds Ratio, CI = Confidence Interval*

*The outcome in the model is a pregnancy with at least one adverse pregnancy outcome (vs. no adverse outcomes).*

*The model includes only those mothers who had no missing data for any key variable included in the model, therefore the number of pregnancies is fewer than the number of women enrolled in the study.*

**Table S7:** Demographic and clinical characteristics of all live newborns and stillbirths diagnosed with invasive Group B streptococcus (iGBS) disease.

| **Characteristic (n=35)** | **Frequency (%)** |
| --- | --- |
| **Identified from** |  |
| Birth Cohort | 5 (14.3) |
| Active Surveillance | 30 (85.7) |
| **Onset of disease** |  |
| Early Onset Disease (EOGBS) | 29 (82.9) |
| Late Onset Disease (LOGBS) | 4 (11.4) |
| Stillbirths | 2 (5.7) |
| **Gestational age at birth** |  |
| Term | 22 (62.9) |
| Preterm | 11 (31.4) |
| Unknown | 2 (5.7) |
| **Sex** |  |
| Male | 17 (48.6) |
| Female | 18 (51.4) |
| **Birthweight** |  |
| LBW (<2500 gr) | 14 (40.0) |
| Normal BW (>2500 gr) | 19 (54.3) |
| Unknown | 2 (5.7) |
| **Maternal HIV Status** |  |
| Living with HIV | 3 (8.6) |
| Not living with HIV | 26 (74.3) |
| Unknown | 6 (17.1) |
| **Outcome of admission among livebirths (n=33)** |  |
| Discharged home alive | 27 (77.1) |
| Died during admission | 6 (18.2) |

**Table S8:** Key genotypic characteristics and phenotypic antimicrobial susceptibility of colonizing and invasive Group B streptococcus (GBS) isolates. CPS: capsular polysaccharide; ST: Sequence Type; CC: Clonal Complex alp: Alpha-like protein; TET: tetracycline; MLSB: macrolide-lincosamide-streptogramin B; MIC: minimum inhibitory concentration; pos: positive; neg: negative; NA: Not available; NT: Not typed; S: susceptible; R: resistant.

**Figure S1**: A. Serotype distribution among the main GBS clonal identified in maternal colonisation and invasive GBS disease isolates; B. Alpha-like protein gene distribution among the main GBS clonal complexes identified in maternal colonisation and invasive GBS disease isolates. GBS: Group B Streptococcus; CC: Clonal Complex; alp: Alpha-like protein; NT: Non-typed.

**Alt text:** Bar plots showing serotype and alpha-like protein gene distribution among major GBS clonal complexes in maternal colonization and invasive disease isolates.

**Figure S2:** Alpha family protein gene distribution among the GBS capsular polysaccharide serotypes identified in maternal colonisation and invasive GBS disease isolates. GBS: Group B Streptococcus; CPS: capsular polysaccharide; alp: Alpha-like protein.

**Alt text**: Bar plot showing the distribution of alpha-like protein genes among GBS capsular polysaccharide serotypes in maternal colonization and invasive disease isolates.

**Figure S3**: A. Presence of MLSB resistance genes among the main GBS clonal complexes identified in maternal colonisation and invasive GBS disease isolates; B. Presence of MLSB resistance genes among the GBS CPS serotypes identified in maternal colonisation and invasive GBS disease isolates. GBS: Group B Streptococcus; CC: Clonal Complex; MLSB: macrolide-lincosamide-streptogramin B; NT: Non-typed.

**Alt text**: Bar plots showing macrolide resistance genes by clonal complex (A) and serotype (B) in maternal colonization and invasive disease isolates.

**Figure S4**: A. Anti-CPS IgG concentrations among infant case patients with EOGBS and controls for serotype Ia; B. Anti-CPS IgG concentrations among infant case patients with EOGBS and controls for serotype III; C. Anti-CPS IgG concentrations among infant case patients with EOGBS and controls for aggregated serotypes. Each point represents an individual sample. Yellow points indicate cord serum. Red points indicate infant serum collected during the acute phase of the disease. CPS: Capsular Polysaccharide; IgG: Immunoglobulin G. EOGBS: Early-onset Group B Streptococcal neonatal disease (0-6 days).

**Alt text**: Dot and box plots showing anti-Capsular Polysaccharide IgG concentrations in infant case patients with early-onset Group B Streptococcal neonatal disease (0-6 days) and controls for (A) Serotype Ia, (B) Serotype III, and (C) Aggregated serotypes. Yellow dots represent cord serum, and red dots represent infant serum from the acute phase.

Additional References

1 Essential Maternal and Newborn Clinical Care Guidelines for Uganda THE REPUBLIC OF UGANDA MINISTRY OF HEALTH. 2022.

2 Qiagen. DNeasy ® Blood & Tissue Handbook W W W. Q I A G E N. C O M. 2006.

3 Page AJ, De Silva N, Hunt M, *et al.* Robust high-throughput prokaryote de novo assembly and improvement pipeline for Illumina data. *Microb Genom* 2016; **2**: e000083.

4 Zerbino DR, Birney E. Velvet: Algorithms for de novo short read assembly using de Bruijn graphs. *Genome Res* 2008; **18**: 821.

5 Boetzer M, Henkel C V., Jansen HJ, Butler D, Pirovano W. Scaffolding pre-assembled contigs using SSPACE. *Bioinformatics* 2011; **27**: 578–9.

6 Boetzer M, Pirovano W. Toward almost closed genomes with GapFiller. *Genome Biol* 2012; **13**: 1–9.

7 Seemann T. Prokka: rapid prokaryotic genome annotation. *Bioinformatics* 2014; **30**: 2068–9.

8 Pruitt KD, Tatusova T, Brown GR, Maglott DR. NCBI Reference Sequences (RefSeq): current status, new features and genome annotation policy. *Nucleic Acids Res* 2012; **40**. DOI:10.1093/NAR/GKR1079.

9 Nascimento M, Sousa A, Ramirez M, Francisco AP, Carriço JA, Vaz C. PHYLOViZ 2.0: providing scalable data integration and visualization for multiple phylogenetic inference methods. *Bioinformatics* 2017; **33**: 128–9.

10 Buurman ET, Timofeyeva Y, Gu J, *et al.* A Novel Hexavalent Capsular Polysaccharide Conjugate Vaccine (GBS6) for the Prevention of Neonatal Group B Streptococcal Infections by Maternal Immunization. *Journal of Infectious Diseases* 2019; **220**: 105–15.

11 Esadze A, Grube CD, Wellnitz S, *et al.* Calibration of a serum reference standard for Group B streptococcal polysaccharide conjugate vaccine development using surface plasmon resonance. *npj Vaccines 2023 8:1* 2023; **8**: 1–10.

12 Madhi SA, Anderson AS, Absalon J, *et al.* Potential for Maternally Administered Vaccine for Infant Group B Streptococcus. *N Engl J Med* 2023; **389**: 215–27.
