# Supplementary figures and images for: Epidemiology of Group B Streptococcus: Maternal Colonization and Infant Disease in Kampala, Uganda
Source: Open Forum Infect Dis. 2025 Mar 18;12(4):ofaf167. doi: 10.1093/ofid/ofaf167 (PMC11977330; doi:10.1093/ofid/ofaf167)

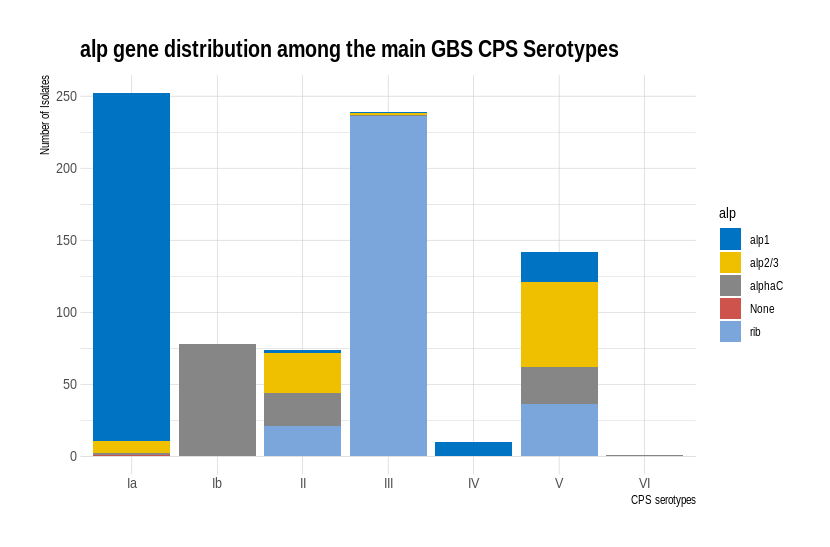

Supplement: ofaf167_Supplementary_Data [file ofaf167_supplementary_data.zip › Figure S2.tif]

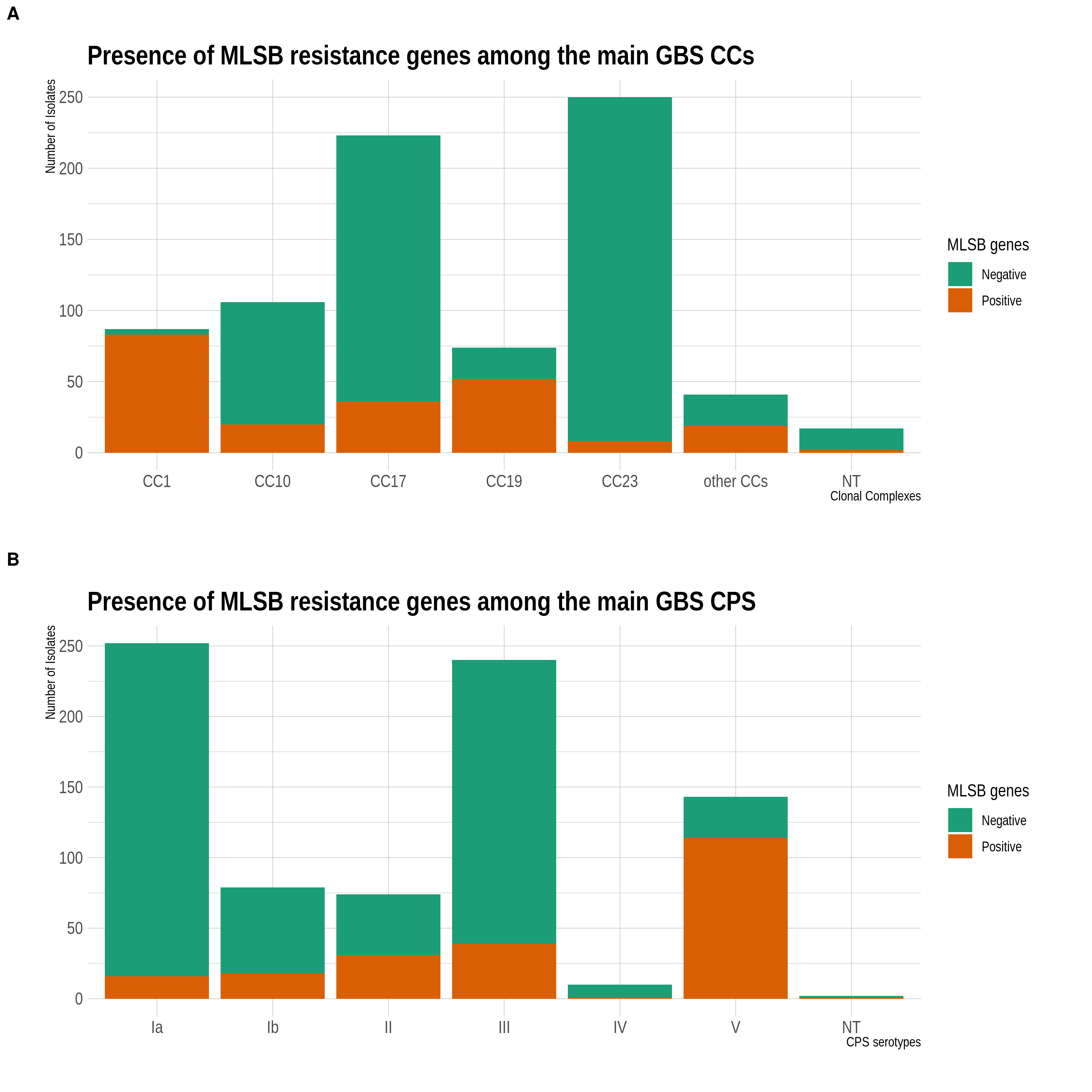

Supplement: ofaf167_Supplementary_Data [file ofaf167_supplementary_data.zip › Figure S3.tif]

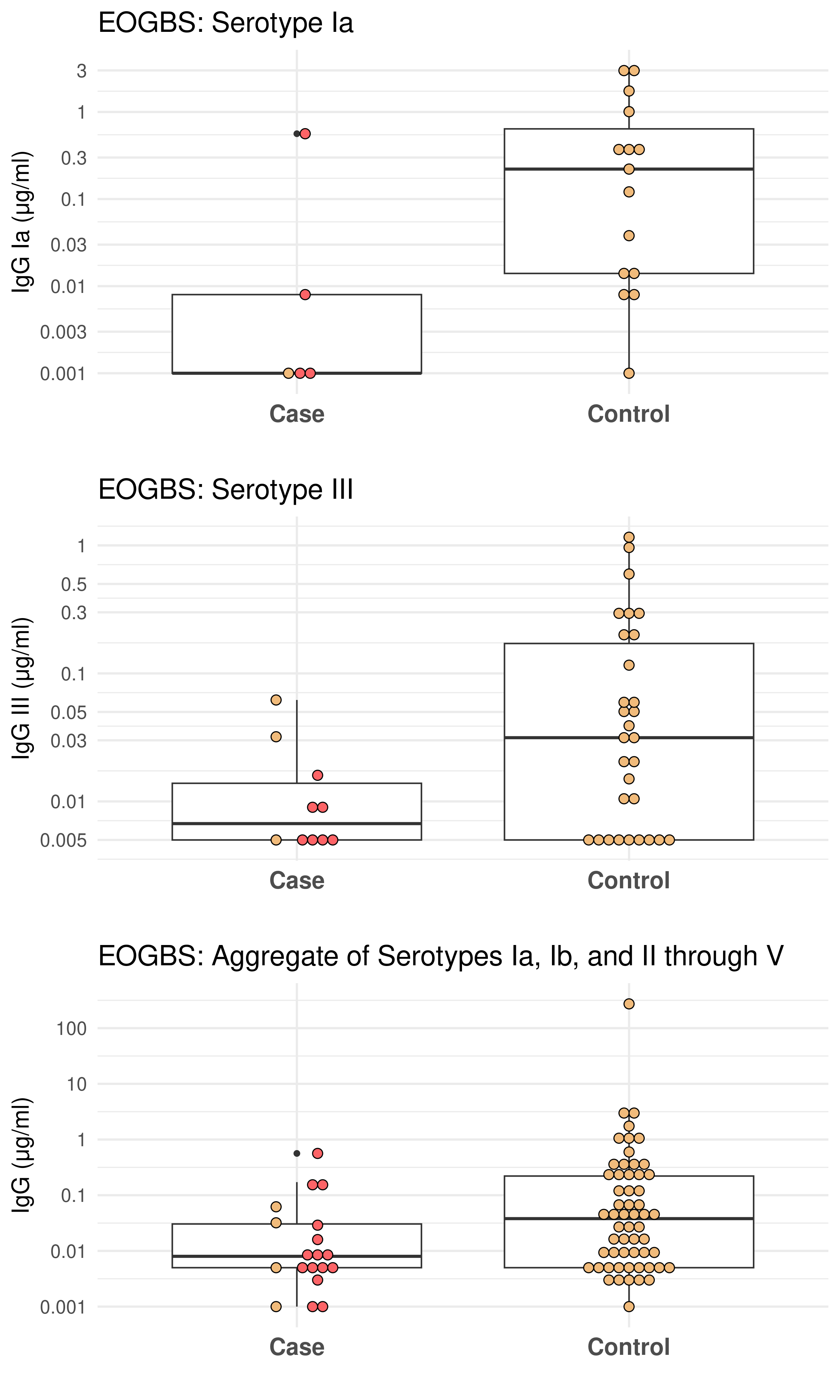

Supplement: ofaf167_Supplementary_Data [file ofaf167_supplementary_data.zip › Figure S4.tif]

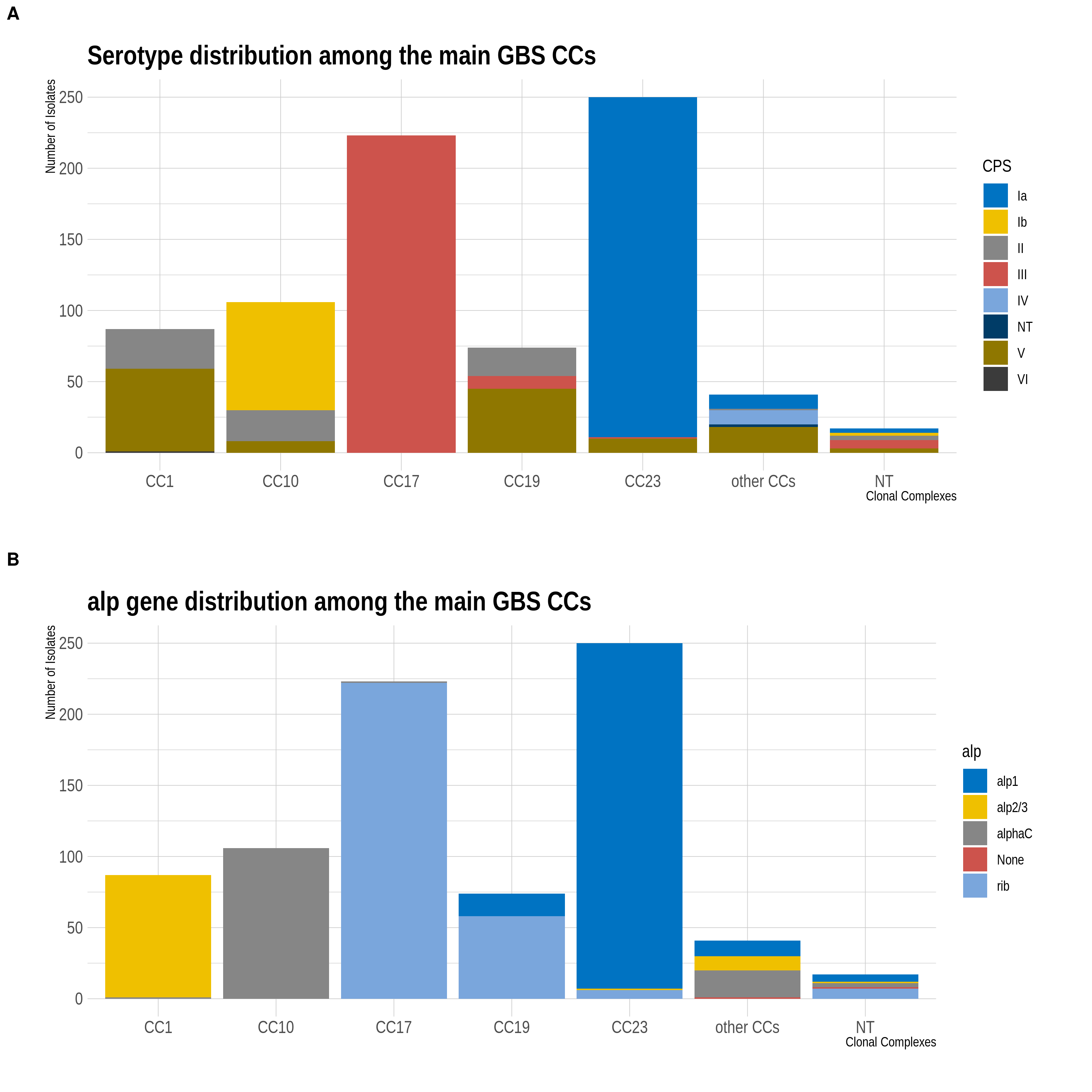

Supplement: ofaf167_Supplementary_Data [file ofaf167_supplementary_data.zip › Figure S1.tif]
